# Burden in Informal Caregivers of Palliative Care Patients With Pressure Injuries: Perceived Social Support and Influencing Factors Care Burden in Palliative Caregivers

**DOI:** 10.1111/jep.70514

**Published:** 2026-06-30

**Authors:** Nadiye Barış Eren, Mehmet Ağaslan

**Affiliations:** ^1^ Department of Nursing, Faculty of Health Sciences Tarsus University Mersin Türkiye

**Keywords:** caregiver burden, caregivers, family caregivers, palliative care, pressure ulcer, social support, wounds and injuries

## Abstract

**Aims:**

This study was designed as a descriptive and correlational study to assess the burden experienced by caregivers of palliative care patients with pressure injuries, to identify the factors influencing this burden and to examine the role of perceived social support.

**Methods:**

Data were collected from 138 informal caregivers of palliative care patients with pressure injuries in a state hospital. The Burden Interview and the Multidimensional Perceived Social Support Scale were used to collect data. Descriptive statistics, correlation analyses and hierarchical regression were conducted to identify factors associated with caregiver burden.

**Results:**

Caregivers reported a high level of burden. Lower income status, difficulty experienced during caregiving and negative effects on family and social life were associated with greater burden. Higher perceived social support was associated with lower burden, indicating that social support plays a protective role for caregivers of patients with pressure injuries.

**Conclusion:**

Caregivers of palliative care patients with pressure injuries experience substantial burden influenced by socioeconomic and caregiving‐related factors. Increased perceived social support contributes to reducing this burden and should be considered an essential component of caregiver‐focused interventions.

## 1 Introduction

Pressure injuries are defined as localized skin or subcutaneous tissue damage typically occurring over bony prominences or in areas of contact with medical devices or other equipment [1]. In a global review evaluating the prevalence of pressure injuries in long‐term care settings, 17 studies were included, and the reported prevalence ranged from 3.4% to 32.4% [2]. A study conducted in Turkey following up on 1548 patients who received inpatient treatment for over 24 h reported that pressure injuries developed in 177 patients (11.43%) [3].

Pressure injuries frequently occur in patients receiving treatment in palliative care units, where risk factors such as older age, prolonged bed rest, immobility and malnutrition are common [4]. The occurrence of pressure injuries can lead to prolonged hospital stays, increased treatment/care costs and a negative impact on the quality of life of patients and caregivers [5, 6]. A study evaluating pressure injuries upon admission to a palliative care unit reported that 83.9% of 566 patients were at risk of pressure injuries [7]. Another study investigating the incidence of pressure injuries in palliative care patients determined that the rate was 12.7%. In addition, palliative care patients were found to have a higher risk of pressure injuries compared to the general population [8].

Palliative care patients frequently experience prolonged hospital stays due to extended treatment processes; as a result, both patients and caregivers face various challenges. Pressure injuries, as one such challenge, can hinder the progress of successful treatment and care [9]. Thus, caregivers bear considerable responsibility for the wound monitoring and care of patients with pressure injuries admitted to palliative care units [5].

To prevent the development of pressure injuries—or mitigate their severity—it is necessary to implement a combination of measures such as position changes, skin care, maintaining adequate and balanced nutrition, preventing fluid loss, wound care and monitoring, mobility and psychosocial support [4]. The continuous and multidimensional nature of these care needs may place an additional physical, emotional and social burden on caregivers. Particularly in palliative care patients, the complex and prolonged caregiving process may make it difficult for caregivers not only to meet the patients' daily needs but also to maintain care in a regular and sustainable manner. Therefore, social support emerges as an important resource that may help caregivers manage this process more effectively. Social support may contribute to strengthening caregivers emotionally, reducing feelings of loneliness during the caregiving process, sharing care responsibilities and alleviating caregiver burden. From a theoretical perspective, the Stress Process Model suggests that caregiver burden arises from the interaction of primary stressors directly related to caregiving tasks and secondary stressors affecting other areas of life [10]. In addition, Social Support Theory, particularly the buffering hypothesis, proposes that social support can mitigate the negative effects of stress by acting as a protective resource for individuals under challenging conditions [11]. In this context, perceived social support may play a critical role in reducing caregiver burden among individuals providing care for patients with pressure injuries.

A study conducted among palliative care patients reported that caregivers had a high level of burden, and 90% of them received support while providing care [5]. Similarly, many studies have emphasized that social support plays an important role in improving caregivers' quality of life and reducing caregiver burden [9, 10, 11, 12, 13, 14].

In light of this information, examining caregiver burden, the factors affecting this burden, and the role of perceived social support among caregivers of palliative care patients with pressure injuries is important for the development of both patient‐ and caregiver‐focused interventions. Although some studies have investigated caregiver burden and perceived social support among caregivers of palliative care patients [5, 15, 16, 17, 18], evidence specifically focusing on caregivers of patients with pressure injuries remains limited, particularly in terms of studies that examine both the factors influencing caregiver burden and the role of perceived social support together. Accordingly, this study aimed to determine the burden levels of caregivers of palliative care patients with pressure injuries, the factors affecting this burden and the role of perceived social support.

### 1.1 Research Questions

1. What is the level of care burden experienced by informal caregivers of palliative care patients with pressure injuries?
2. What factors influence the care burden experienced by informal caregivers of palliative care patients with pressure injuries?
3. Does perceived social support influence the care burden experienced by informal caregivers of palliative care patients with pressure injuries?

## 2 Materials and Methods

### 2.1 Study Design

This study was conducted as a cross‐sectional analytical study among informal caregivers of patients with pressure injuries admitted to the palliative care unit of a state hospital in Turkey. The study was reported in accordance with the STROBE (Strengthening the Reporting of Observational Studies in Epidemiology) guidelines for observational studies.

### 2.2 Study Population

This study included informal caregivers of patients with pressure injuries admitted to the palliative care units of a secondary‐level district state hospital in Turkey. The hospital had two palliative care units with a total capacity of 24 beds. The units predominantly provided care for patients with advanced dementia/Alzheimer's disease and end‐stage oncology conditions requiring palliative care. The institution did not function as a referral centre. The sample size was calculated using the formula for unknown populations [19]. In the calculation, the *t* value was set at 1.96 for a 95% confidence level, the margin of error (*d*) was accepted as 0.05 and the probability of occurrence (*p*) was accepted as 0.10 based on the pressure injury frequency reported in previous studies [3, 8], ensuring that the sample size estimation was appropriate for the target population of this study; accordingly, the probability of non‐occurrence (*q*) was accepted as 0.90. The minimum required sample size was calculated as 138 (*n* = *t* ^2^ *pq*/d^2^ = (1.96)^2^(0.10)(0.90)/(0.05)^2^ = 138.29). During the data collection process, eligible caregivers who met the inclusion criteria and agreed to participate were consecutively recruited until the target sample size was reached. A total of 138 eligible informal caregivers were enroled in the study, and none declined participation. All questionnaires were complete, no missing data were identified, and no cases were excluded from the final analysis. A convenience sampling method was used, and eligible caregivers who met the inclusion criteria and agreed to participate were consecutively recruited during the data collection period until the target sample size was reached.

### 2.3 Inclusion Criteria

Informal caregivers of patients with pressure injuries admitted to the palliative care units were eligible for inclusion if they were aged 18 years or older, had no verbal communication problems and agreed to participate voluntarily in the study.

### 2.4 Exclusion Criteria

Professional/formal caregivers, individuals younger than 18 years, those with verbal communication problems that could interfere with data collection and those who declined to participate were excluded from the study.

### 2.5 Data Collection

This study included individuals who met the inclusion criteria and provided both verbal and written consent. The research was conducted in person at the state hospital palliative care unit between August 2023 and February 2024 through questionnaires and interviews with the caregivers. The questionnaires were collected by the researchers upon completion. Each interview lasted an average of 10–15 min.

Data were collected using three instruments: a descriptive characteristics form, the Burden Interview (BI) and the Multidimensional Perceived Social Support Scale (MSPSS).

The descriptive characteristics form was developed by the researcher following a review of the literature [5, 15, 16]. The form consisted of 16 questions covering the caregivers’ sociodemographic characteristics and patient‐ and pressure injury‐related characteristics. The caregivers’ sociodemographic variables included age, gender, education level, marital status, presence of chronic disease, employment status and monthly income level. The patient‐ and pressure injury‐related variables included pressure injury stage, duration of pressure injury (days), daily care duration (hours), total care duration (months), prior pressure injury training, degree of relationship to the care recipient, difficulty experienced in providing care, the effect of caregiving on family and social life and the status of receiving assistance in providing care.

The BI scale was developed by Zarit et al. [20] to assess the burden experienced by caregivers. A Turkish adaptation and validity–reliability study of the scale was published by İnci and Erdem [21]. The scale has a unidimensional structure consisting of 22 items; each item has a five‐point Likert‐type rating scale ranging from 0 to 4 points with the options ‘never’, ‘rarely’, ‘sometimes’, ‘often’ and ‘almost always’. The total score ranges from 0 to 88, with a score increase indicating an increase in the burden experienced by the caregiver. Cutoff scores are as follows: 0–20 points = ‘no or very low care burden’; 21–40 points = ‘mild–moderate care burden’; 41–60 points = ‘moderate–severe care burden’; and 61–88 points = ‘severe care burden’. In the Turkish validity–reliability study, the Cronbach's *⍺* coefficient of the scale was 0.95 [21]; in this study, this value was calculated as 0.94.

The MSPSS was developed by Zimet et al. [22]. The validity–reliability study of the Turkish adaptation was conducted by Eker and Arkar [23]. A revised version of the scale was subsequently developed by Eker et al. [24]. The scale consists of 12 items, with a three‐subscale structure. It includes three subgroups based on the source of support: family, friends and a special person; each group consists of four items. The subgroups are defined as family (3, 4, 8, 11), friends (6, 7, 9, 12) and a special person (1, 2, 5, 10), respectively. However, in the present study, evaluation was based solely on the total score, in line with the research objective. Each item on the scale is rated on a seven‐point Likert scale, ranging from 1 to 7. The total score obtainable ranges from 12 to 84, with high scores indicating that the individual perceives a high level of social support. In the Turkish validity–reliability study, the Cronbach's *⍺* coefficient for the total score of the scale was 0.86 [24]; in this study, this value was calculated as 0.95.

### 2.6 Ethical Approval

Ethical approval for this study was obtained from the Ethics Committee on 4 July 2023 (Decision No.: 2023/32). The study was conducted at a state hospital, and institutional permission was obtained from the hospital administration on 10 August 2023 (Decision No.: 60). Informed consent was ensured by providing participants with detailed information about the study, emphasizing voluntary participation to respect autonomy and maintaining confidentiality of the data obtained. Both written and verbal consent were obtained from participants who agreed to take part in the study. The study was conducted in accordance with the principles of the Declaration of Helsinki to protect participants' rights throughout the research.

### 2.7 Data Analysis

Data analysis was performed using the SPSS Statistics for Windows Version 25.0 (IBM Corp., 2017) software package. Descriptive statistics, including counts, percentages, means and standard deviations, were calculated. The normality of continuous variables was assessed using skewness and kurtosis coefficients. An independent samples *t*‐test was used for comparisons between two groups; one‐way analysis of variance was used for comparisons between three or more groups. The Pearson's correlation coefficient was calculated to determine the relationships between variables. Hierarchical multiple linear regression analysis was applied to identify the significant variables predicting care burden. Before conducting the hierarchical multiple linear regression analysis, regression assumptions were evaluated. Multicollinearity was assessed using tolerance and variance inflation factor (VIF) values, independence of errors was examined using the Durbin–Watson statistic and residual normality and influential observations were evaluated using standardized residuals, studentized residuals, the normal P–P plot and Cook's distance values. The independent variables included in the regression models were determined based on their conceptual relevance and prior literature on caregiver burden and perceived social support, as well as results from pairwise comparisons and correlation analyses. Care burden (total BI score) was used as the dependent variable. The analysis was conducted in three stages: in the first model, employment status, monthly income level and the presence of chronic diseases were included as control variables. The second model included the difficulty of providing care and the impact of caregiving on family and social life. The third model included the perceived social support score. Significance was determined at *p* < 0.05.

## 3 Results

Examining the distribution of participants by demographic characteristics showed that 76.8% of caregivers (average age 54.38 ± 12.74 years) were female, 60.9% had completed primary education, 83.3% were married and 60.1% had no chronic illnesses. A total of 73.9% of caregivers were not employed, with 57.2% earning a monthly income lower than their expenses. The average duration of pressure injuries in patients receiving care was 50.99 ± 54.16 days, with 34.1% classified as Stage I pressure injuries. Notably, 65.9% of caregivers had received no training on pressure injuries. Regarding kinship, 39.9% of caregivers were the patients' children. The average daily care time was 13.39 ± 8.26 h; the total care time was 32.94 ± 55.59 months on average. Caregivers' family and social lives were affected in 78.3% of cases, with 84.8% receiving assistance while providing care (Table 1).

**Table 1:** Descriptive characteristics of informal caregivers (*N* = 138).

Characteristics	*n*	%
Gender		
Female	106	76.8
Male	32	23.2
Education level		
Illiterate	15	10.9
Primary education	84	60.9
High school and above	39	28.2
Marital status		
Married	115	83.3
Single	23	16.7
Presence of chronic disease		
Yes	55	39.9
No	83	60.1
Employment status		
Employed	36	26.1
Unemployed	102	73.9
Monthly income level		
Income < Expenses	79	57.2
Income ≥ Expenses	59	42.8
Stage of the care recipient's pressure injury		
Stage 1	47	34.1
Stage 2	34	24.6
Stage 3	39	28.3
Stage 4	18	13.0
Prior pressure injury training		
Yes	47	34.1
No	91	65.9
Degree of relationship to the care recipient		
Spouse	36	26.1
Child	55	39.9
Daughter‐in‐law/son‐in‐law	30	21.7
Parent/sibling	17	12.3
Difficulty experienced in providing care		
Yes	117	84.8
No	21	15.2
Effect of providing care on family and social life		
Yes	108	78.3
No	30	21.7
Status of receiving assistance in providing care		
Yes	117	84.8
No	21	15.2
Age (X̄ ± SD: 54.38 ± 12.74)		
Duration of pressure injury (days) (X̄ ± SD: 50.99 ± 54.16)		
Daily care duration (h) (X̄ ± SD: 13.39 ± 8.26)		
Total care duration (months) (X̄ ± SD: 32.94 ± 55.59)		

Abbreviations: *n* = frequency, SD = standard deviation, X̄ = mean, % = percentage.

Comparison of caregiver characteristics with average care burden scores revealed statistically significant differences based on the presence of chronic illness, unemployment, low income levels, difficulty in providing care and impact of caregiving on family and social life (*p* < 0.05). No statistically significant differences were found based on gender, educational level, marital status, training on pressure injuries or receiving assistance while providing care (*p* > 0.05) (Table 2).

**Table 2:** Comparison of caregivers' descriptive characteristics and BI scores (*N* = 138).

Characteristics	*n*	BI (X̄ ± SD)	Mean difference (95% CI)	Test	*p*
Gender					
Female	106	58.31 ± 18.74	−0.28 (−7.89 to 7.32)	*t*: −0.073	0.94
Male	32	58.59 ± 20.17			
Education level					
Illiterate	15	61.86 ± 18.50	—	F: 0.474	0.62
Primary education	84	58.67 ± 19.10			
High school and above	39	56.38 ± 19.24			
Marital status					
Married	115	58.08 ± 19.44	−1.73 (−10.35 to 6.87)	*t*: −0.392	0.69
Single	23	59.82 ± 16.98			
Presence of chronic disease					
Yes	55	63.25 ± 23.76	8.10 (1.69 to 14.52)	*t*: 2.500	< 0.05*
No	83	55.14 ± 16.82			
Employment status					
Employed	36	52.55 ± 19.28	−7.87 (−15.06 to −0.68)	*t*: −2.166	< 0.05*
Unemployed	102	60.43 ± 18.57			
Monthly income level					
Income < Expenses	59	50.33 ± 18.64	−14.04 (−20.08 to 8.00)	*t*: ‐4.728	< 0.01*
Income ≥ Expenses	79	64.37 ± 17.05			
Prior pressure injury training					
Yes	47	57.00 ± 18.95	−2.08 (−8.85 to 4.68)	*t*: −0.610	0.54
No	91	59.08 ± 19.10			
Difficulty experienced in providing care					
Yes	117	60.78 ± 17.74	15.38 (7.30 to 24.36)	*t*: 8.215	< 0.01*
No	21	44.95 ± 20.64			
Effect of providing care on family and social life					
Yes	108	63.96 ± 15.58	25.69 (19.24 to 32.15)	*t*: 7.873	< 0.01*
No	30	38.26 ± 16.98			
Status of receiving assistance in providing care					
Yes	117	57.41 ± 19.53	−6.35 (−15.22 to 2.52)	*t*: −1.415	0.15
No	21	63.76 ± 15.04			

Abbreviations: BI = burden interview, F = one‐way ANOVA, *n* = frequency, SD = standard deviation, *t* = independent sample *t*‐test, X̄ = mean.

*Note:* *p < 0.05.

The correlation analysis between age, pressure injury duration, total care duration and daily care duration and BI scores also revealed no statistically significant differences. A moderate, negative and statistically significant relationship was found between the total MSPSS score and BI score (*r* = −0.556, *p* < 0.001) In addition, all MSPSS subscales were significantly and negatively correlated with caregiver burden: family support (*r* = −0.456, *p* < 0.001), friend support (*r* = −0.555, *p* < 0.001) and significant other support (*r* = −0.566, *p* < 0.001) (Table 3).

**Table 3:** Correlation analysis of age, duration of pressure injury, total and daily care duration and MSPSS scores with caregiver burden.

	Age	Duration of pressure injury	Daily care duration	Total care duration	Family subscale	Friends subscale	A special person subscale	MSPSS
BI	*r* = 0.116 *p* = 0.17	*r* = −0.045 *p* = 0.59	*r* = 0.004 *p* = 0.96	*r* = −0.010 *p* = 0.91	*r* = −0.456 *p* < 0.001	*r* = −0.555 *p* < 0.001	*r* = −0.566 *p* < 0.001	*r* = −0.556 *p* < 0.001*

Abbreviations: BI = burden interview, MSPSS = multidimensional scale of perceived social support, *r* = Pearson's correlation analysis.

* *p* < 0.05.

Regarding the responses to the caregiving burden scale, approximately half of the caregivers reported a severe care burden (48.6%), with 32.6% reporting a moderate–severe care burden, 16.6% reporting a mild–moderate care burden and 2.2% reporting a no or very low care burden. The mean total BI score was 58.23 ± 18.98, and the mean total MSPSS score was 42.15 ± 22.94. Among the MSPSS subscales, the mean scores were 11.39 ± 8.70 for family support, 13.40 ± 7.83 for friend support and 17.31 ± 7.84 for support from a significant other (Table 4).

**Table 4:** Caregivers' mean scale scores (*N* = 138).

BI	*n*	%
No or very low care burden (0–20)	3	2.2
Mild–moderate care burden (21–40)	23	16.6
Moderate–severe care burden (41–60)	45	32.6
Severe care burden (61–88)	67	48.6

	**X̄ ± SD**	**Min–Max**
BI (total score)	58.23 ± 18.98	0–80
MSPSS (total score)	42.15 ± 22.94	12–84
Family subscale	11.39 ± 8.70	4–28
Friends subscale	13.40 ± 7.83	4–28
A special person subscale	17.31 ± 7.84	4–28

Abbreviations: BI = burden interview, Max = maximum, Min = minimum, MSPSS = multidimensional scale of perceived social support, *n* = frequency, SD = standard deviation, X̄ = mean, % = percentage.

Model 1 revealed a significant relationship between income status (*β* = 0.32, *p* < 0.001) and BI score. The model explained 15.8% of the total variance (*R* ^2^ = 0.158; adjusted *R* ^2^ = 0.139, *p* < 0.001). Model 2 included the variables of difficulty in providing care (*β* = 0.16, *p* < 0.05) and the impact of providing care on family and social life (*β* = 0.48, *p* < 0.001). As a result of this inclusion, the income status variable, which was significant in the previous model, retained its significance (*β* = 0.17, *p* < 0.05). Model 2 explained 42.4% of the total variance (*R* ^2^ = 0.424; adjusted *R* ^2^ = 0.402, *p* < 0.001) and provided an additional 26.6% increase in explained variance compared with Model 1 (Δ*R* ^2^ = 0.266). The MSPSS score was added to Model 3; a significant negative relationship between this variable and the BI score was determined (*β* = −0.34, *p* < 0.001). In this model, the previously significant variable of difficulty in providing care lost significance (*β* = 0.10, *p* > 0.05); whereas the variable of caregiving affecting family and social life retained significance (*β* = 0.37, *p* < 0.001). Model 3 explained 51.1% of the total variance (*R* ^2^ = 0.511; adjusted *R* ^2^ = 0.489, *p* < 0.001) and provided an additional 8.8% increase in explained variance compared with Model 2 (Δ*R* ^2^ = 0.088). Regression diagnostics supported the suitability of the hierarchical multiple linear regression model. In the final model, tolerance values (0.748–0.900) and VIF values (1.111–1.337) indicated no multicollinearity, the Durbin–Watson value (1.871) supported independence of errors and the normal P–P plot suggested approximate normality of residuals. In addition, Cook's distance values (maximum = 0.053), as well as standardized and studentized residuals, indicated no influential outliers.

## 4 Discussion

This study aimed to examine the burden levels among caregivers of palliative care patients with pressure injuries, the factors influencing this burden and the role of perceived social support.

Approximately half of the caregivers experienced severe care burdens (Table 4). This finding is supported by studies reporting severe care burdens among caregivers of palliative care [17] and home care [25] patients. However, some studies report low levels of care burden among caregivers of patients receiving palliative care and primary healthcare [26, 27]. The severe burden among caregivers observed in this study may be due to the included caregivers providing care to palliative patients with the exacerbating factor of pressure injuries.

In this study, caregivers with lower income levels reported heavier care burdens (Table 5). In contrast, Bekdemir and Ilhan [28] and Tay et al. [29] reported that income levels did not affect care burden, while Bilgehan and İnkaya [5] reported that caregivers with higher income levels had greater care burdens. This difference may be attributable to variations in socioeconomic levels of the study locations. Supporting the findings of the present study, several studies have reported that caregivers with low income levels experience higher care burdens [30, 31, 32]. This suggests that limited economic resources may increase care burden by preventing necessary material needs from being met during the care process. Furthermore, the fact that the monthly income level continues to have a significant effect on care burden independently of other factors highlights its importance as a determinant of care burden.

**Table 5:** The hierarchical linear regression analysis for caregiver burden (*N* = 138).

Items	Model 1			Model 2			Model 3		
	B (95% CI)	*β*	*t*	B (95% CI)	*β*	*t*	B (95% CI)	*β*	*t*
Presence of chronic disease (ref: Yes)	4.15 (−2.25 to 10.56)	0.10	1.28	3.30 (−2.04 to 8.65)	0.08	1.22	1.14 (−3.87 to 6.17)	0.03	0.45
Employment status (ref: Employed)	2.77 (−4.46 to 10.01)	0.06	0.75	2.06 (−3.99 to 8.12)	0.04	0.67	3.17 (−2.45 to 8.79)	0.07	1.11
Monthly income level (ref: Income < Expenses)	12.40 (5.91 to 18.89)	0.32	3.77*	6.81 (1.21 to 12.41)	0.17	2.40*	5.06 (−0.15 to 10.29)	0.13	1.91
Difficulty experienced in providing care (ref: Yes)				8.58 (1.45 to 15.70)	0.16	2.38*	5.47 (−1.23 to 12.18)	0.10	1.61
Effect of providing care on family and social life (ref: Yes)				22.38 (16.13 to 28.63)	0.48	7.08*	17.12 (10.96 to 23.28)	0.37	5.49*
MSPSS							−0.28 (−0.39 to −0.16)	−0.34	−4.84*
*F*		8.36*			19.40*			22.83*	
*R* ^2^		0.15			0.42			0.51	
Adjusted *R* ^2^		0.13			0.40			0.48	
Δ*R* ^2^					0.26			0.08	

Abbreviations: *β* = standardized regression coefficient, *F* = *F*‐test value, MSPSS = multidimensional scale of perceived social support, ref. = reference group, *R* ^2^ = coefficient of determination (explained variance), Δ*R* ^2^ = change in *R* ^2^, *t* = *t*‐test value.

* *p* < 0.05 (two‐tailed).

This study found that caregiver employment status had no significant effect on care burden. Tay et al. [29] reported similar findings. However, Bekdemir and Ilhan [28] reported that unemployment increased the burden of care. This suggests that the factors affecting the burden of care may not be limited to employment status.

In this study, caregivers' health problems did not have a significant effect on care burden. In contrast, Bekdemir and Ilhan [28] reported that health problems in caregivers significantly increased care burden. This difference is believed to stem from the fact that caregivers in the cited study provided care to patients who were bedridden and required home care services.

Difficulties encountered while providing care and the impact on the caregiver's family and social life play a significant role in determining the burden of care. The findings of the present study reveal that caregivers experiencing such difficulties report a higher burden of care. Öner and Karabulutlu [33] and Parvizi and Ay [34] reported similar results. The high level of dependency of palliative care patients, the protracted treatment periods and the complex nature of their care can negatively impact the family and social lives of caregivers, increasing the burden of care.

This study found moderate levels of perceived social support by caregivers. Previous studies have reported that social support is the primary facilitator of care and ensures successful execution of the care process [9, 15]. In this study, the perception of social support was found to have a significant mitigating effect on care burden. The 8.8% increase in explanatory power with the addition of social support indicates that this is an important variable in predicting care burden which should not be overlooked in treatment and care processes, and that comprehensive assessments of individuals with weak perceptions of social support are critical. Furthermore, this finding parallels previous research highlighting the role of social support in alleviating caregiver burden [12, 33, 35, 36, 37, 38, 39].

## 5 Clinical Implication

Integrating caregiver‐focused social support strategies into palliative care services is essential, as higher perceived social support significantly reduces caregiver burden. Enhancing the social and emotional support provided to caregivers can improve their well‐being and contribute to better overall palliative care outcomes. Embedding structured caregiver support within palliative care policies and clinical guidelines may strengthen service delivery and promote sustainable caregiving practices.

From a clinical perspective, the findings of this study suggest that healthcare professionals, particularly nurses working in palliative care units, should systematically assess caregiver burden and perceived social support during routine care. Structured caregiver support programs, including education on pressure injury care, emotional support interventions and guidance on sharing caregiving responsibilities, may help reduce caregiver burden. Previous studies have also emphasized that caregiver‐focused interventions and social support mechanisms play a critical role in improving caregiver outcomes and reducing burden [36, 39]. In addition, providing counselling and guidance to caregivers to help them cope with caregiving challenges may contribute to reducing caregiver burden [33]. Integrating caregiver‐focused support strategies into palliative care services, such as counselling services and multidisciplinary support systems, may contribute to improving both caregiver well‐being and the overall quality of care provided to patients.

## 6 Limitations

The study has several limitations. First, as the data were collected from a single state hospital, the generalizability of the findings is limited. Second, since only caregivers who agreed to participate were included, the possibility of selection bias cannot be completely excluded. Third, as the data were collected using self‐report instruments, there is a potential risk of bias related to participants' perceptions and responses. In addition, information on patients was limited, and important clinical characteristics of pressure injuries, such as patient age, educational status, length of hospital stay, number of wounds, anatomical location, presence of infection and level of care dependency, were not assessed. Therefore, the findings should be interpreted with caution in terms of both generalizability and clinical detail.

Future studies should include the evaluation of these clinical characteristics and may consider examining additional variables such as caregiver resilience, coping strategies and psychological well‐being to better understand factors influencing caregiver burden.

## 7 Conclusion

This study was conducted to examine the levels of care burden in caregivers of palliative care patients with pressure injuries, the factors influencing this burden and the role of perceived social support. Participants experienced high levels of care burden, with approximately half belonging to the ‘heavy care burden’ category. Hierarchical regression analysis identified low income, experiencing difficulty while providing care, and negative impact on family and social life as significant variables increasing care burden. The key finding was a strong negative relationship between perceived social support and care burden, indicating that high social support significantly reduces care burden. This highlights the need to develop approaches focusing not only on patients but also on maintaining the physical and psychological well‐being of caregivers during the palliative care process. Complications such as pressure injuries increase the already complex care requirements, thus increasing the burden on caregivers and underscoring the need to strengthen social support mechanisms. These findings highlight the importance of integrating caregiver‐focused support strategies, including routine assessment of caregiver burden and enhancement of perceived social support, into palliative care practices.

## Author Contributions

**Nadiye Barış Eren:** conceptualization, investigation, formal analysis, methodology, writing – original draft preparation, writing – review and editing. **Mehmet Ağaslan:** investigation, writing – original draft preparation, formal analysis.

## Funding

The authors have nothing to report.

## Ethics Statement

Ethical approval was obtained from Tarsus University Non‐Interventional Clinical Research Ethics Committee committee (date: 4 July 2023; decision no.: 2023/32), and institutional permission was granted by the hospital (date: 10 August 2023; decision no.: 60). Informed consent was ensured by providing participants with information about the study, respecting autonomy by emphasizing voluntary participation and maintaining confidentiality regarding the information obtained. Both written and verbal consent were obtained from willing participants. The Helsinki Declaration of Human Rights was followed to protect individuals' rights throughout the study.

## Conflicts of Interest

The authors declare no conflicts of interest.

## Data Availability

The data that support the findings of this study are available from the corresponding author upon reasonable request. No permission was required to reproduce material from other sources.
